# Supplementary material for: Synthetic lethality between HER2 and transaldolase in intrinsically resistant HER2-positive breast cancers
Source: Nat Commun. 2018 Oct 15;9:4274. doi: 10.1038/s41467-018-06651-x (PMC6189078; doi:10.1038/s41467-018-06651-x)
Supplement: Supplementary file 1 — Supplementary Information [file 41467_2018_6651_MOESM1_ESM.pdf]

SUPPLEMENTARY INFORMATION

Synthetic lethality between HER2 and transaldolase in intrinsically resistant HER2-positive breast cancers

Ding et al.

Supplementary Figure 1

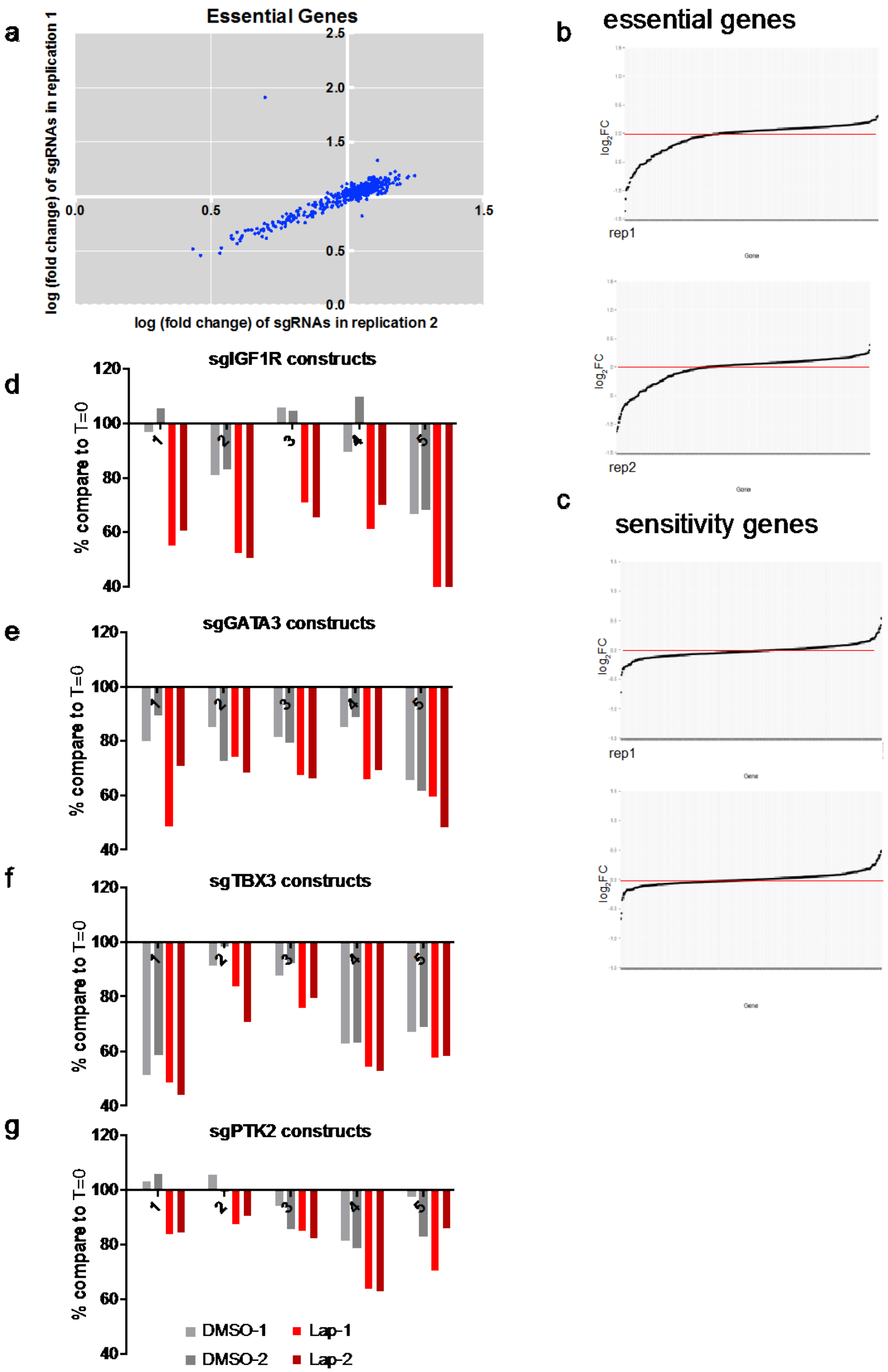

Supplementary Figure 1.

Quality control and genes identified from CRISPR/Cas9 profiling.

(a) Log_2 (fold change) of each gene from two biological replicates. Fold change was calculated as the mean of 5 sgRNA concentration at T=2weeks/T=0 for each lapatinib-treated replicate. The linear alignment of dots presenting each gene indicated consistency between the two replicates.

(b-c) Depletion of each gene is shown in Log_2 (fold change) of T=2weeks-vehicle-treated/T=0 as essential for cell survival (b); and Log_2 (fold change) of T=2weeks-lapatinib-treated/T=0 as mediating sensitivity to lapatinib (c).

(d-g) Percentages of individual sgRNA constructs targeting the indicated genes in samples treated with lapatinib for 2 weeks compared to DMSO treatment conditions.

a

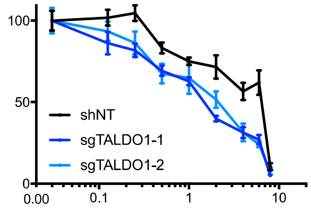

d

e

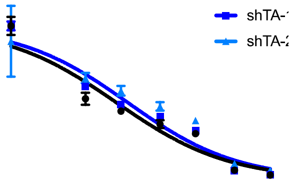

Supplementary Figure 2.

CRISPR/Cas9 screening hit validation.

(a-d) Percentages of viable cells after treatment with the indicated concentrations of lapatinib for 7 days, as measured by Cell-Titer-Glo. MDA-MB-361 cells were infected with lentiviral particles carrying non-targeting control or sgRNA constructs targeting the indicated genes (n=4).

(e) Percentages of viable cells after treatment with the indicated concentrations of lapatinib for 7 days, as measured by Cell-Titer-Glo. MDA-MB-453 cells were infected with lentiviral particles carrying non-targeting control or shTA (n=4).

(f-g) Percentages of viable cells transfected with shNT or shTA after treatment with the indicated concentrations of lapatinib for 5 days, as measured by Cell-Titer-Glo. Top, western blots for TA and tubulin show shRNA knockdown efficiency (n=4). IC50s were calculated using the Prism curve fit (variable-slope) default program. Error bars, SD.

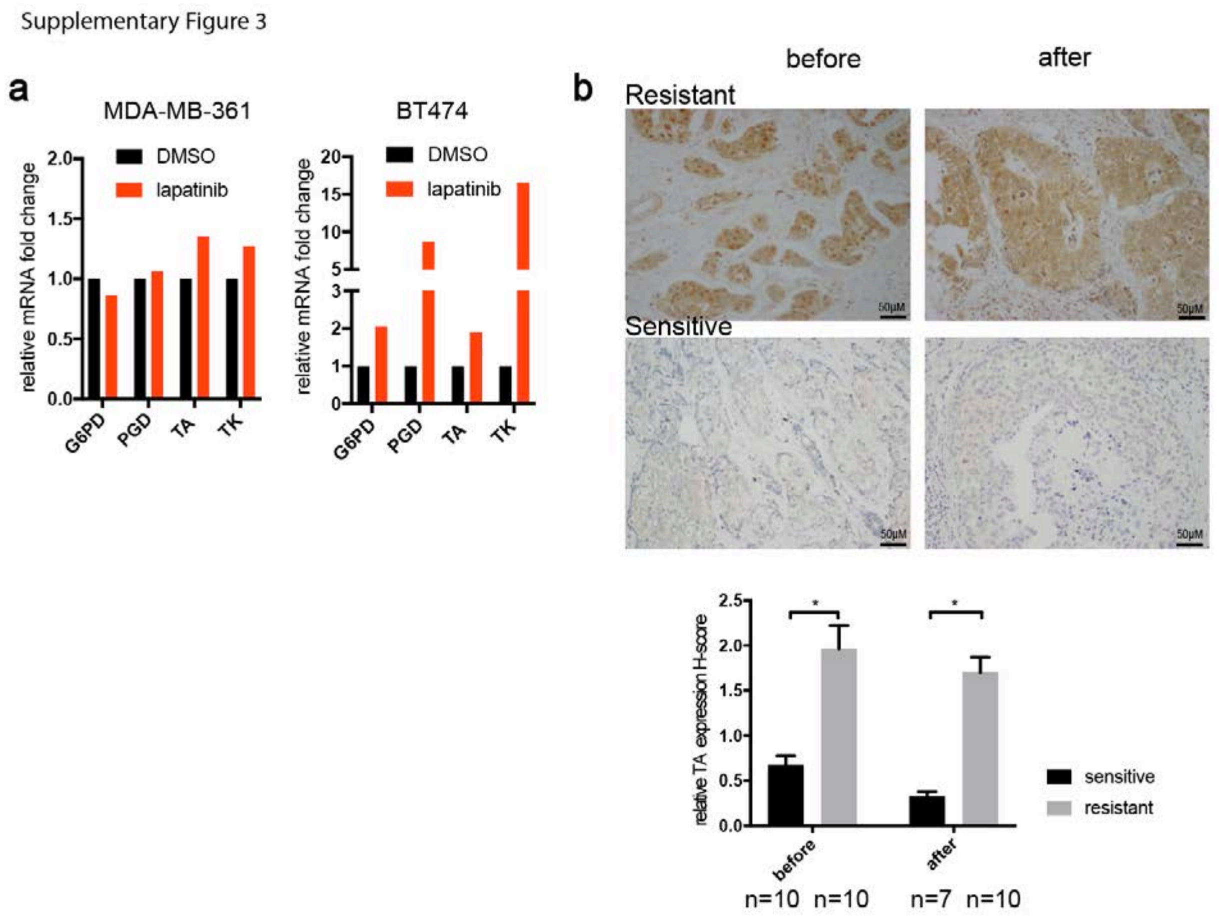

Supplementary Figure 3.

PPP enzyme expression in cell lines and TA expression in patient tissues

(a) Relative mRNA expression of MDA-MB-361 cells and BT-474 cells treated with DMSO or 1 μM lapatinib for 24 hours (n=2).

(b) Representative images of TA immunostaining in resistant (PD+SD) and sensitive (PR+pCR) breast cancer tissues. ‘Before’ samples were obtained from HER2-positive breast cancers by core-needle biopsy prior to therapy; ‘after’ samples were obtained from surgically removed tumor tissues. ‘Before’, n=10 resistant tumors (PD+SD), n=10 sensitive tumors (PR+pCR) biopsied collected; ‘after’, all resistant tumors and 7 out of 10 sensitive tumors were surgically removed after neoadjuvant therapy. Quantification of TA immunohistochemistry in resistant and sensitive tumors. Error bars, SD; * *P*<0.001, student t test; PR, partial remission; PD, progressive disease; SD, stable disease; pCR, pathological complete response.

Supplementary Figure 4

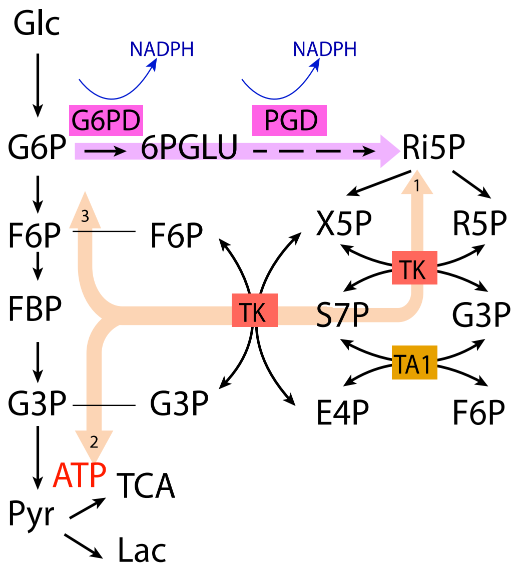

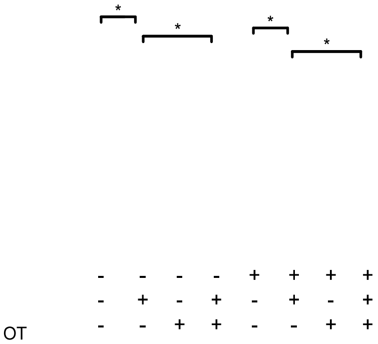

Supplementary Figure 4.

TK is essential for cell survival but does not synergize with HER2 inhibition.

(a) Schematic of PPP metabolites and possible directions of metabolic flux. Arrows indicate 3 different metabolic outflow scenarios.

(b) Fluorescence signal strength reflecting cell viability as measured by Cell-Title-Glo. MDA-MB-361 cells were infected with shNT or shTA, then treated with 10 μM lapatinib and/or 2 mM oxythiamine (OT) for 6 days (n=4). Error bars, SD; * $P<0.01$, student t test.

(c) Cell viability as measured by CellTiter-Glo. Cells carrying the indicated shRNA constructs were treated with DMSO or 4 μM lapatinib and assayed on days 0, 3, 6, and 9 (n=4).

(d) Percentages of individual sgRNA targeting TKT constructs in indicated samples compared to time point zero T=0.

(e) Kaplan-Meier relapse-free survival (RFS) analysis of HER2-amplified breast cancer patients with low-quartile TK expression (black) and the rest (red).

Supplementary Figure 5

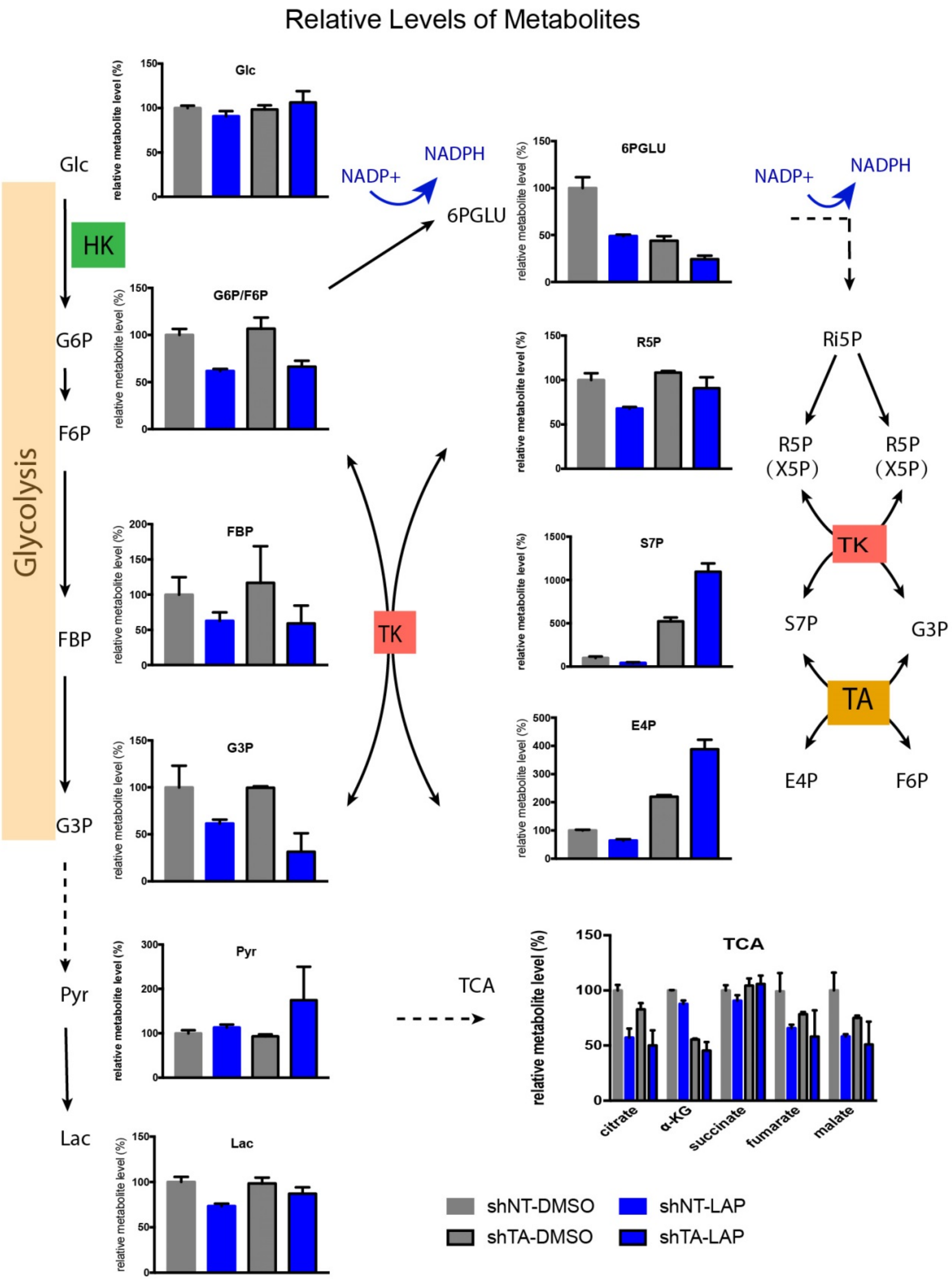

Supplementary Figure 5:

Metabolic profiling of cells treated with lapatinib and/or deficient in TA. Total levels of metabolite mapped in the pathway. Abbreviations: Glc, glucose; G6P, glucose-6-phosphate; 6PGLU, 6-phosphogluconate; R5P, ribulose-5-phosphate; F6P, fructose-6-phosphate; FBP, fructose-1,2-biphosphate; G3P, glyceraldhyde-3-phosphate; S7P, sedoheptulose-7-phosphate; E4P, erythrose-4-phosphate; Pyr, pyruvate; Lac, lactate; TCA, tricarboxylic acid. Error bars, SD (n=3).

ycolysis and PPP

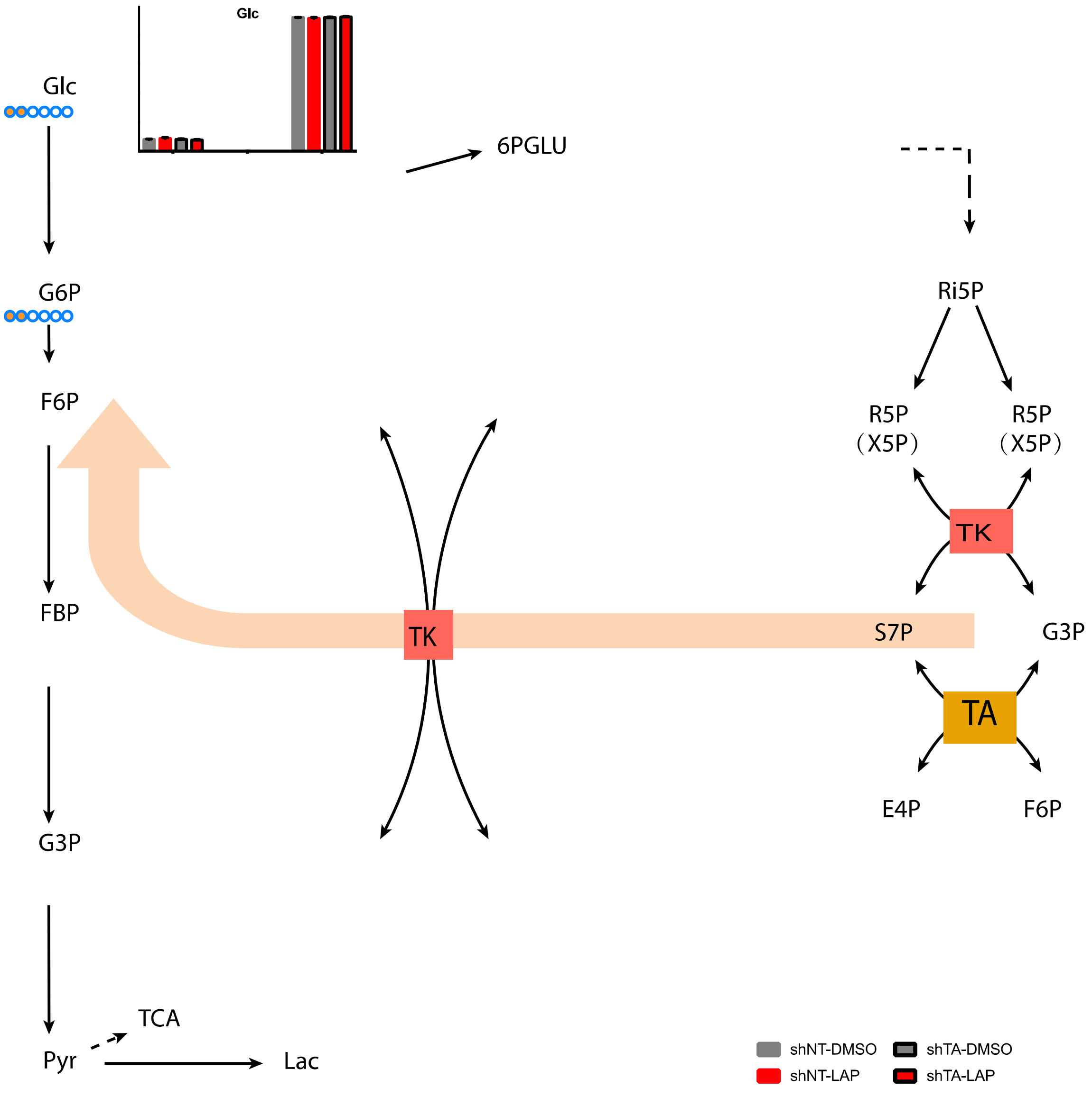

Supplementary Figure 6:

Metabolic profiling of cells treated with lapatinib and/or deficient in TA. Fractions of single isotopes of each metabolite mapped into metabolic pathway. Schematic depicting the fate of labeled ^{13}C (orange-filled) from 1,2-$^{13}C_2$-labeled glucose in glycolytic [51] and PPP (gray) intermediates. Abbreviations: Glc, glucose; G6P, glucose-6-phosphate; 6PGLU, 6-phosphogluconate; R5P, ribulose-5-phosphate; F6P, fructose-6-phosphate; FBP, fructose-1,2-biphosphate; G3P, glyceraldhyde-3-phosphate; S7P, sedoheptulose-7-phosphate; E4P, erythrose-4-phosphate; Pyr, pyruvate; Lac, lactate; TCA, tricarboxylic acid. Error bars, SD (n=3).

Supplementary Figure 7

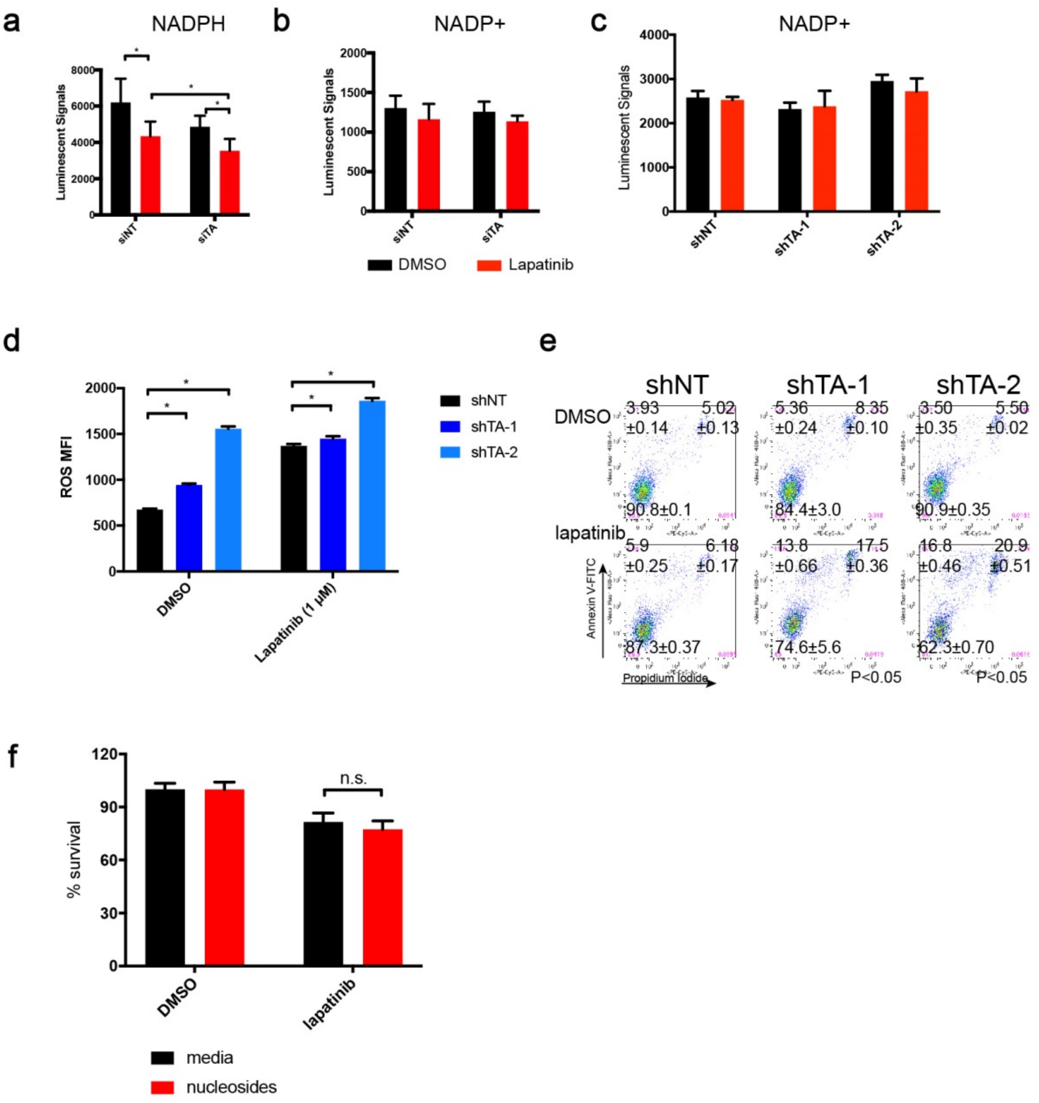

Supplementary Figure 7:

MDA-MB-453 cells are sensitive to combined TA and HER2 inhibition.

(a-b) Relative concentrations of NADPH and NADP+ were detected using a bioluminescent assay. MDA-MB-453 cells were transfected with siNT or siTA and treated with 1 μM lapatinib for 48 hrs (n=4).

(c) Relative concentrations of NADP+ of MDA-MB-361 cells.

(d-e) MDA-MB-453 cells stably expressing shNT or shTA were treated with 1 μM lapatinib for 48 hrs. (d) Cellular ROS levels were measured by Cell Rox deep red flow

cytometry staining. Mean fluorescence intensity±SEM was calculated using FlowJo (n=3). (e) Percentages of apoptotic cells were detected by Annexin V-FITC and PI staining. Numbers indicate the percentages of total evaluated cells±SEM (n=3). P-value shows Q1 and Q2 percentages with student t-test.
(f) Relative survival of MDA-MB-361 cells transfected with siTA, then treated with lapatinib (1 μM) and/or nucleosides for 3 days (n=4).
Error bars, SD; * $P<0.01$, student t test; n.s., not significant.

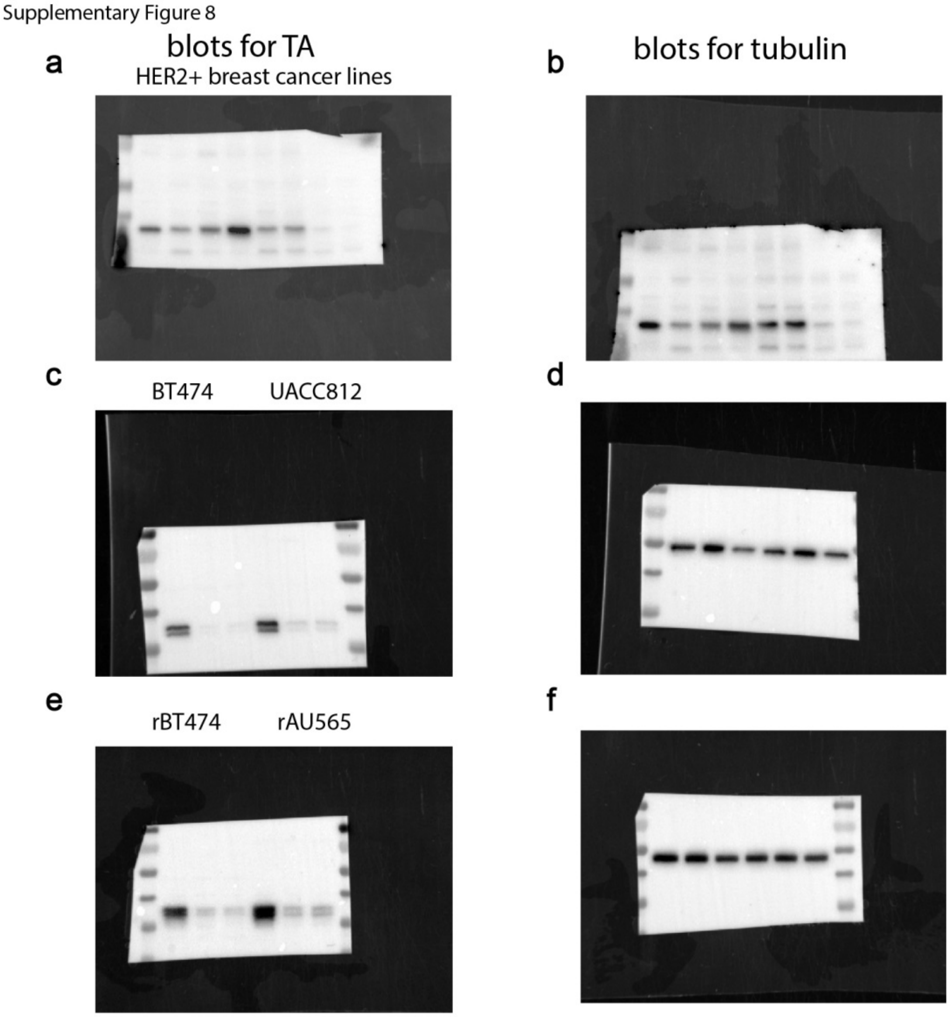

Supplementary Figure 8: Original pictures of western blots.

(a-b) Whole blot image of HER2 positive breast cancer cell lines. Scrapped images were shown in Fig. 3a.
(c-d) Whole blot image of BT474 and UACC812 cells stably expressing shNT or shTA constructs. Scrapped images were shown in Fig. 2d.
(e-f) Whole blot image of rBT474 and rAU565 cells stably expressing shNT or shTA constructs. Scrapped images were shown in Fig. 2d.
